# Challenges in establishing optimal pediatric palliative care at the university hospital in Slovenia

**DOI:** 10.1007/s00431-023-04806-7

**Published:** 2023-01-21

**Authors:** Jakob Meglič, Ajda Lisec, Dušanka Lepej, Tanja Loboda, Sara Bertok, Petra Lešnik Musek, Ivana Kreft Hausmeister, Majda Oštir, Tehvida Ponjević, Anamarija Meglič

**Affiliations:** 1 University Medical Centre, Ljubljana, Slovenia; 2 Pediatric Palliative Team, University Children’s Hospital, University Medical Centre, Ljubljana, Slovenia

**Keywords:** Pediatric palliative care, Implementation, Life-limiting disease, Life-threatening condition, Quality of children’s life

## Abstract

The integration of pediatric palliative care (PPC) should become a standard of care for all children with life-limiting and life-threatening illnesses. There are many barriers and misperceptions in pediatrics which hinder the early implementation of PPC. The aim of the study was to design starting points for the establishment of accessible PPC with early involvement of patients in a tertiary-level children’s hospital. An intervention, presentation, and discussion on PPC were offered by the hospital PPC team to all employees in the hospital. A total of 237 participants (physicians 30.4%, nurses 49.4%, psychologists 8.4%, and others) completed a questionnaire before and after the intervention. The personnel’s knowledge, self-assessment of their ability to perform PPC, attitude to participate in PPC, and their awareness and understanding of the need for PPC were evaluated. The results were analyzed using Pandas and SciPy libraries in Python. The knowledge, awareness, and attitude of the physicians, nurses, and other professionals improved significantly after the intervention. However, the self-assessment of their ability to perform PPC did not increase. Previous experience with the death of a patient has proven to be a stimulus for self-initiative in acquiring knowledge in PPC and was linked with a better attitude and higher awareness of the need for PPC.

*Conclusions*: More education and practical work tailored to the different professional profiles are needed, with adjustments for specific subspecialist areas, especially where patients could be included in early PPC. Although additional studies are needed, we identified the main directions for the further implementation of PPC in clinical practice in our setting.

**Supplementary Information:**

The online version contains supplementary material available at 10.1007/s00431-023-04806-7.

**Table:** 

**What is Known:** *•* *Every child and adolescent living with a life-limiting or life-threatening condition should receive pediatric palliative care (PPC) to alleviate suffering and enhance their quality of life. There exists a plethora of recognized barriers to the effective implementation of palliative care, specifically PPC. These barriers are often connected to the emotional burden of requesting PPC. Early identification and inclusion of patients is important for improving PPC in hospital settings. Finding strategies to overcome the barriers is crucial for improving the well-being and improving the quality of life of the patients and their families.* *•* *Early identification is only possible with a high level of knowledge and understanding of PPC among healthcare professionals. In a hospital setting where there are interdisciplinary palliative care teams, the inclusion is still only possible if all staff are capable of recognizing patients in need of PPC and are willing to start the process. Since most healthcare education systems only recently included PC into the healthcare curriculum, most of the professionals currently working in hospitals are only educated to the extent of self-initiative.*	
**What is New:** *•* *To bridge the existing gap in knowledge, the hospital PC team organized an intervention, presentation, and discussion on PPC, which was offered to all employees in the hospital who are in contact with patients. The personnel’s knowledge, self-assessment of their ability to perform PPC, attitude to participate in PPC, and their awareness and understanding of the need for PPC were evaluated. These four categories have not been tested together before. The knowledge, awareness, and attitude of the physicians, nurses, and other professionals improved significantly after the intervention. All the profiles that work together in a team were evaluated simultaneously for the first time.* *•* *The self-assessment of their ability to perform PPC did not increase—in fact, it decreased. This was unexpected, as existing literature establishes a link between education and quality of PC. Previous experience with the death of a patient has proven to be a stimulus for self-initiative in acquiring knowledge in PPC and was linked with a better attitude and higher awareness of the need for PPC. We re-established the importance of education and practical work tailored to the different professional profiles, with adjustments for specific subspecialist areas, especially where patients could be included in early PPC. Although additional studies are needed, we identified the main directions for the further implementation of PPC in clinical practice in our setting.*	

## Background

According to current guidelines, every child and adolescent living with a life-limiting or life-threatening condition should receive pediatric palliative care (PPC) to alleviate suffering and enhance their quality of life [1–3]. The integration of PPC should become a standard of care for all [2, 4, 5].

There are many barriers and misperceptions in pediatrics which hinder the implementation of PPC, among which the most important is undoubtedly the emotional labor involved in requesting palliative care (PC) [6–8]. Some professionals are concerned about the term “palliative care” itself, feeling its interpretation by families may be negative [9]. Physicians fail to discuss prognosis, because of fear of upsetting the patient and damaging the therapeutic relationship. [10]. Therefore, it is crucial that other professionals, e.g., nurses, who are in closest contact with the family, also know the benefits of palliative care (PC) and are experienced in identifying new patients [11–13].

For the effective implementation of PPC in any environment, it is important to understand what PPC is and what it is not [9, 14]. To improve PC in hospital settings, research and practice have shown that it is first necessary to enable the early identification of patients in need of PC [15]. If healthcare professionals have only a basic knowledge of PC, patient involvement may be late, often only in the process of dying [16–18].

The interdisciplinary hospital PC team at the Children’s Hospital offers PC consultation, help in advance care planning, and advice in providing support to families [19, 20]. Although the hospital PC team is helpful, it is necessary for the medical staff to be able to identify a patient who would benefit from early PC and to request the hospital PC team’s cooperation [21, 22]. Consequently, they all help to ensure that children are exposed to a minimum amount of suffering [5, 23].

In Slovenia, most children with life-limiting or life-threatening conditions are identified at the University Tertiary Children’s Hospital in Ljubljana. A few years ago, a hospital PC team was established, consisting of two pediatric pulmonologists, two pediatric neurologists, a pediatric nephrologist, a clinical geneticist, two registered nurses, and two clinical psychologists. The members of the team are all trained in PPC and have more than 15 years of work experience in their respective fields. In the 2 years between the team’s inception and the intervention, the team has managed PPC of 70 patients, coordinating different healthcare providers involved in their care, as well as offering full-time support to the families. Soon after the team was established, many obstacles in the implementation of PC were recognized. Team members decided to raise awareness of PC’s importance in all departments of the hospital. We considered the recommendations of Eddy et al. [24]: all members of the team participated in the design and implementation of a questionnaire and intervention.

With the study, the hospital PC team aimed to offer all staff members a pediatric-specific orientation in PC appropriate to their roles and responsibilities, and to attract the largest possible number of employees to participate. We also wanted to ascertain the state of the medical staff: knowledge, self-assessment of their ability to perform, and attitude towards and awareness of the importance of PC. The objective, based on the findings of the project, was to design starting points for further education and the establishment of accessible, optimal PPC with early involvement of different patients from all departments in the hospital.

## Methods

The medical and paramedical personnel of the institution were invited by the heads of their departments to an intervention, presentation, and discussion on PPC, as well as a survey before and after the intervention. The purpose of the survey was to evaluate four categories: the personnel’s knowledge, self-assessment of their ability to perform PPC, attitude and willingness to participate in PC, and awareness and understanding of the need for PPC.

The interventions were conducted by the hospital PPC team and carried out between March 2020 and May 2021 in each hospital department. The lectures were specifically designed for the purpose of the intervention by the team members, who are all experienced lecturers. The lectures were taught by them in Slovenian. The intervention consisted of providing definitions as well as posing provocative questions on what we could do to keep the patients from suffering. It consisted of three parts. The first part covered the physician’s view of the benefits of PC in children and adolescents with life-limiting or life-threatening conditions, and how we can alleviate distress with individually tailored treatment for any of them. In the second part, a nurse demonstrated actions that are easy to perform in the scope of nursing care and provide great relief to both patients and their parents. In the last part, a psychologist presented PPC from the patient’s perspective of quality of life and their perception of PPC, presenting various coping mechanisms for serious illness in children and adolescents in different stages of development, and what the child’s illness means for the whole family. In all parts, tips were given for better communication with patients, relatives, and within the medical team. An extensive discussion followed at the end of each session. Despite each department receiving a separate presentation, all were of the same content, which was approved by all team members. The lectures were not tested before, to avoid interfering with the results.

The first part of the completely anonymous questionnaire (Supplement 1), designed especially for the project by the hospital PPC team, consisted of demographic questions concerning profession, age, years of experience in pediatrics, experience of a patient dying, and previous participation in PC.

The second part of the questionnaire was aimed at evaluating different areas relating to PC: the healthcare professional’s knowledge (knowledge), self-assessment of their ability to perform PC (self-assessment), attitude and willingness to participate in PC (attitude), and their awareness and understanding of the need for PC (awareness). To quantify subjective preferential opinion, thinking, and feeling in a scientifically accepted, validated, and reliable manner, we used the Likert 5-point symmetrical scale. The questions were formed as statements where participants chose a number between 1 and 5, with number 1 meaning “strongly disagree” and number 5 “strongly agree.” The participants’ mean score of all answers in each of the four categories was calculated and used for further analysis.

The complete anonymity of respondents was ensured in accordance with data protection laws and ethical standards. The results obtained were not linked to any individual or to the department from which data were obtained to prevent the disclosure of participants’ identity.

The results were analyzed using Pandas (version 1.4.2) and SciPy (version 1.8.1) libraries in Python (version 3.10.4; Python Software Foundation, Wilmington, DE, USA).

The Shapiro–Wilk test was used to determine the distribution of data. As most data sets did not follow Gaussian distribution, we used non-parametric tests.

For comparison of results regarding the four categories before and after intervention, the Wilcoxon signed-rank test was used. When comparing different groups regarding knowledge, self-assessment, attitude, and awareness, the Mann–Whitney *U* test was used.

## Results

### Demographics

Among all 292 attendees of the intervention, 237 took the survey before and after the intervention. The breakdown of participants by profession is noted in Tables 1 and 2.

**Table 1:** Number and proportions of different professionals among participants

Broad category	Profession	Number of participants
Medical	Physician	72 (30.4%)
	Nurse	117 (49.4%)
	Psychologist	20 (8.4%)
Paramedical	Physical therapist	4
	Radiology engineer	3
Other	Dietitian	4
	Teacher	8
	Kindergarten teacher	4
	Other	5
		237 (100%)

**Table 2:** Subspecialty breakdown of the participating consultant physicians

	Department												Total
	Emergency	Neonatology	Nephrology	Gastroenterology	Pulmonology	Cardiology	Endocrinology	Hematology-oncology	Allergology	Neurology	Pediatric psychiatry	ICU	
Participating consultant physicians	1	4	5	4	4	3	6	7	4	2	4	1	45

The age structure of participants was as follows: 23.7% (*n* = 55) between 20 and 30 years, 31.9% (*n* = 74) between 31 and 40 years, 20.7% (*n* = 48) between 51 and 60 years, 24.6% (*n* = 57) between 41 and 50 years, and 1.3% (*n* = 3) were older than 60 years.

Among the participants, 35.0% (*n* = 83) had up to 5 years of experience in pediatrics, 16.9% (*n* = 40) had 5 to 15 years of experience, and 46.8% (*n* = 111) had more than 15 years of experience.

**Fig. 1:**
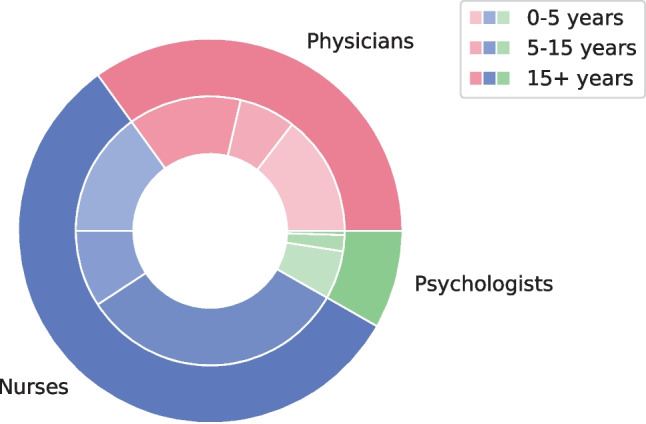
Years of experience of the medical professionals

In the group of medical professionals, 41.7% (*n* = 30) of physicians had less than 5 years of experience in pediatrics, 19.4% (*n* = 14) had 5 to 15 years of experience, and 38.9% (*n* = 28) had more than 15 years of experience. Among nurses, 26.5% (*n* = 31) had less than 5 years of experience in pediatrics, 16.2% (*n* = 19) had 5 to 15 years of experience, and 57.3% (*n* = 67) had more than 15 years of experience. Among psychologists, 55% (*n* = 11) had less than 5 years of experience, 25% (*n* = 5) had 5 to 15 years of experience, and 5% (*n* = 1) had more than 15 years of experience in pediatrics. Fifteen percent (*n* = 3) of psychologists did not answer the question (Fig. 1).

There were 69.2% of participants who had already experienced a patient’s death: among physicians 70.1%, among nurses 78.6%, and among psychologists 35%. There were 46.4% of participants who reported previous participation in PC: among physicians 40.3%, among nurses 64.1%, and among psychologists 15.0%.

### Comparison of all categories before and after the intervention

**Fig. 2:**
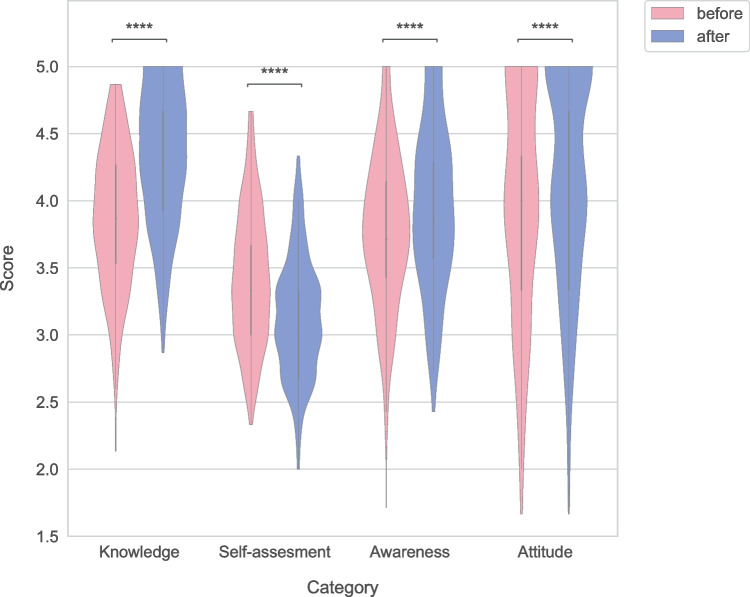
Violin plot of scores for different categories before and after the intervention for all participants. ****p < 0.0001

After the intervention, knowledge was significantly better for all participants combined, but the self-assessment of their ability to perform dropped. Awareness as well as attitude also improved after the intervention (Fig. 2).

### Comparison of categories depending on the experience of a patient’s death before and after the intervention

**Fig. 3:**
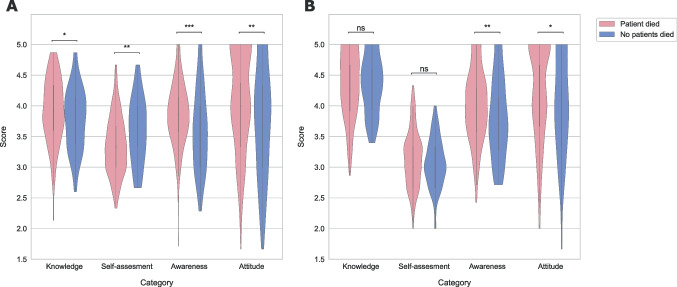
Violin plot of scores for different categories before (**A**) and after (**B**) the intervention depending on whether the participant experienced the death of a patient. NS non-significant, *p < 0.05, **p < 0.01, ***p < 0.001

Before the intervention for all participants combined, knowledge, awareness, and attitude towards PPC were significantly better in participants who had already experienced the death of a patient, but self-assessment was significantly lower than in participants who had not experienced the death of a patient (Fig. 3A). After the intervention, only awareness and attitude were better in participants who had experienced the death of a patient versus participants who had not. Knowledge increased and self-assessment dropped after the intervention but were no longer statistically different between the two groups of participants (Fig. 3B).

We also made comparisons between groups of participants based on age, years of experience, and previous participation in PPC. The only factor that yielded significant differences in all categories was the experience of the death of a patient.

### Comparison of knowledge and self-assessment in the core medical group before and after the intervention

**Fig. 4:**
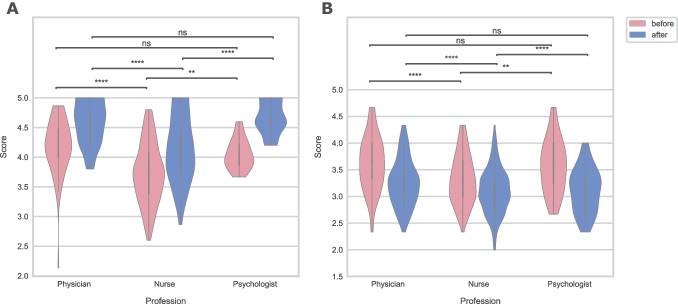
Violin plot of knowledge scores (**A**) and self-assessment scores (**B**) before and after the intervention for physicians, nurses, and psychologists. NS non-significant, **p < 0.01, ****p < 0.0001

A comparison of knowledge and self-assessment among participants of different professions in the medical group before and after the intervention is shown in Fig. 4. Both categories are significantly different in comparing physicians and psychologists versus nurses before and after the intervention.

## Discussion

Given that in our country there is no universal training on PC for healthcare providers, it was expected that the level of knowledge among employees would be relatively low and that education in improving attitude and awareness would be needed for all personnel. At the same time, we realized that for further development, it would be necessary to determine the existing conditions in our hospital. We were not able to find a study in which these categories, including self-assessment of ability to perform PC, attitude, and awareness, were studied in medical professionals of different profiles who work together in a tertiary children’s hospital in different subspecialties. Tsao et al. measured baseline palliative knowledge, attitudes, and self-assessment, but only with physicians from oncology and general adult care [25]. We found some similarities with the study of Newton and Sebbens, which took place in several departments in a tertiary care pediatric facility where the effect of educational sessions on hospital-wide referral rates and provider comfortability after survey was studied. Although the study differed with respect to participant professions, the results are comparable with ours by the positive effect of educational sessions demonstrated. With knowledge and experience, awareness of the need for PC is improved and aversion to work in PC is lessened [26]. Ghoshal et al. evaluated attitude and knowledge along with skills and practice after modules on PPC had been uniformly conducted among healthcare workers in PPC facilities [27]. Physicians and nurses had a better level of knowledge and attitude than social workers and counselors.

It was also clear from the beginning that only a single intervention would not be sufficient for healthcare providers to achieve the satisfactory implementation of PC in hospitals. However, this might prompt the staff to start thinking about PC, become interested in identifying patients who would benefit from PC, and enhance their awareness and attitude of the importance of PC, which may have an important influence on the quality of care they provide [28, 29].

After the intervention, employees answered questions about the PC content more correctly, were more willing to participate in PC, and were more aware of the importance of PC. The result, namely, that the knowledge, attitude, and awareness of all participants increased, is favorable and expected. It is, however, unexpected that the self-assessment of their ability to perform PC did not increase with the intervention.

It is possible that after learning about PC, the participants realized that they were not equipped to provide this care and therefore felt less able to provide it themselves. This, combined with the increase in their awareness and attitude scores, might lead them to seek further education more actively, attending additional lectures and practical workshops, which is a desired outcome.

The only studied factor that yielded significant differences in all categories was the experience of the death of a patient. Apparently, the experience of a patient dying is an important driver for acquiring knowledge, improving attitude, and understanding the importance of PC, but not for feeling the ability to perform it, which is interesting. It was surprising to learn that those participants who had experienced the death of a patient before the intervention expressed feeling less able to perform PC compared to the participants who had not had this experience. Perhaps, the latter were not aware of the complexity of PC and the emotional burden.

The results of a comparison of self-assessment in the two groups of participants after the intervention, which showed that the difference in the self-assessment of their ability to perform PPC after the intervention had disappeared, despite having decreased in both groups, may suggest an explanation for such an outcome. The intervention revealed the difficulty of working with seriously ill children and possibly discouraged the participants, which is the opposite of what we wanted to achieve. Another possibility is that lecturers overemphasized the work of the hospital PC team, causing participants to misunderstand that PC was mainly the obligation of the hospital PC team and not of all employees. Though it is undoubtedly important for the hospital PC team to offer consultation, it should bring home the message that the departmental medical teams need to provide PPC to patients themselves in their daily work.

We can self-critically assess that this intervention alone may not improve the implementation of PC in our environment to the desired extent. It is, however, optimistic that the intervention has helped to increase the participants’ knowledge, attitude, and awareness of the importance of PC. The results showing a decrease in self-assessment of ability to perform PC after the intervention present a clear direction for further research and intervention: what are the reasons for the feeling of inability to perform, also by additionally defining the feelings of those who have already had the experience of a patient dying, and later designing interventions to help overcome these reservations.

The interventions were designed to come closer to the different healthcare professionals’ profiles within each department. They were carried out for all professionals in one department at a time to show them that the work of different profiles is equally important when they work as a medical team in PPC.

Knowledge is difficult to evaluate [30]. Comparing knowledge between different profiles of professionals is especially demanding. Most of the questions in the knowledge category were general statements about what PC is and what it is not. Both physicians and psychologists scored better than nurses in knowledge, which surprised us, especially because the significant difference remained even after education. We infer that our intervention was not sufficiently tailored to the needs of different profiles. The level of knowledge was an insight into the extent of the incorrect notion that PC is simply caring for a dying patient. Even though knowledge increased after the intervention, self-assessment worsened. Since nurses tend to have more constant contact with patients and their families and are sometimes easier to approach with questions, it is crucial they have the knowledge and feel empowered to give advice and talk about early involvement in PPC. We realized that it might be difficult for nurses to identify patients who would benefit from early PPC, since their knowledge scores were lower and they had a significantly greater feeling of inadequate knowledge, when compared to other professionals [12–15, 21, 31–34].

There are several limitations to our study because there is no standardized education material about PC for healthcare providers in our country. No similar research with different professional profiles included in the same tertiary-level pediatric hospital has been found with which we could compare our results. Finally, the survey was designed specifically for the intervention and has not been validated in other environments.

## Conclusions

The knowledge, awareness, and attitude of physicians, nurses, and other professionals in the tertiary-level pediatric hospital improved significantly after the presentation and discussion on PPC provided by the hospital PC team. Previous experience of a patient dying has proven to be a stimulus for self-initiative in acquiring knowledge in PC and improving attitude and awareness towards PC.

We believe that the intervention presents an important first step of PPC implementation. Non-palliative care professionals might be more willing to consult the hospital PC team and talk about PC with their patients, even if they do not feel confident enough to provide it themselves, resulting in more frequent and timely referrals.

In further efforts to improve the implementation of PC in our environment, there are many challenges. More education and practical work tailored to the different professional profiles are needed, and different educational components for training practical skills (e.g., roleplaying) and other adjustments should be used, especially for the subspecialist areas where patients could be included in early PC.

## Supplementary Information

Below is the link to the electronic supplementary material.

Supplementary file1 (DOCX 325 KB)

Supplementary file2 (PDF 104 KB)

## Abbreviations

PC: Palliative care
PPC: Pediatric palliative care
