# Supplementary material for: Challenges in establishing optimal pediatric palliative care at the university hospital in Slovenia
Source: Eur J Pediatr. 2023 Jan 21;182(3):1393–401. doi: 10.1007/s00431-023-04806-7 (PMC10023644; doi:10.1007/s00431-023-04806-7)
Supplement: Supplementary file 1 — Supplementary file1 (DOCX 325 KB) [file 431_2023_4806_MOESM1_ESM.docx]

# **Challenges in establishing optimal pediatric palliative care at the university hospital in Slovenia**

Jakob Meglič^1^* (0000-0003-1686-8165), MD, Ajda Lisec^1^* (0000-0001-5463-3901), MD, Dušanka Lepej^2^, MD, Tanja Loboda^2^ MD, Sara Bertok^2^ , MD, Petra Lešnik Musek^2^,PsyD, PhD, Ivana Kreft Hausmeister^2^, PsyD, PhD, Majda Oštir^2^, RN, MSc, Tehvida Ponjević^2^,RN, Anamarija Meglič^2^ (0000-0002-1273-690X), MD, PhD

^1^ University Medical Centre, Ljubljana, Slovenia

^2^ Pediatric Palliative Team, University Children’s Hospital, University Medical Centre, Ljubljana, Slovenia

*These authors contributed equally to this work.

Corresponding author: Ajda Lisec^1^, lisecajda@gmail.com

# Abstract

**Purpose:** The integration of PPC should become a standard of care for all children with life-limiting and life-threatening illnesses. There are many barriers and misperceptions in pediatrics which hinder the early implementation of PPC. The aim of the study was to design starting points for the establishment of accessible PPC with early involvement of patients in a tertiary-level children’s hospital.

**Methods:** An intervention, presentation, and discussion on PPC were offered by the hospital PPC team to all employees in the hospital. 237 participants (physicians 30.4 %, nurses 49.4%, psychologists 8.4%, and others) completed a questionnaire before and after the intervention. The personnel’s knowledge, self-assessment of their ability to perform PPC, attitude to participate in PPC, as well as their awareness and understanding of the need for PPC, were evaluated. The results were analyzed using Pandas and SciPy libraries in Python.

**Results:** The knowledge, awareness and attitude of the physicians, nurses and other professionals improved significantly after the intervention. However, the self-assessment of their ability to perform PPC did not increase. Previous experience with the death of a patient has proven to be a stimulus for self-initiative in acquiring knowledge in PPC and was linked with a better attitude and higher awareness of the need for PPC.

**Conclusions:** More education and practical work tailored to the different professional profiles are needed, with adjustments for specific subspecialist areas, especially where patients could be included in early PPC. Although additional studies are needed, we identified the main directions for the further implementation of PPC in clinical practice in our setting.

**Keywords:** pediatric palliative care, implementation, life-limiting disease, life-threatening condition, quality of children’s life

# Statements and declarations

The authors have no relevant financial or non-financial interests to disclose.

## Ethics approval

This is an observational study of willing medical professionals, hence no ethical approval was sought.

## Consent to participate

Informed consent was obtained from all individual participants included in the study.

Author Contributions

All authors contributed to the study conception and design. Material preparation and data collection were performed by Dušanka Lepej, Tanja Loboda, Sara Bertok, Petra Lešnik Musek, Ivana Kreft Hausmeister, Majda Oštir and Tehvida Ponjević. The analysis was performed by Jakob Meglič and Ajda Lisec. The first draft of the manuscript was written by Anamarija Meglič, Jakob Meglič and Ajda Lisec, who also commented on previous versions of the manuscript. All authors read and approved the final manuscript.

# What is Known-What is New

## What is Known

- Every child and adolescent living with a life-limiting or life-threatening condition should receive pediatric palliative care (PPC) to alleviate suffering and enhance their quality of life. There exists a plethora of recognized barriers to effective implementation of palliative care, and specifically PPC. These barriers are often connected to the emotional burden of requesting PPC. Early identification and inclusion of patients is important for improving PPC in hospital settings. Finding strategies to overcome the barriers is crucial for improving the well-being and improving the quality of life of the patients and their families.
- Early identification is only possible with a high level of knowledge and understanding of PPC among healthcare professionals. In a hospital setting where there are interdisciplinary palliative care teams, the inclusion is still only possible if all staff is capable of recognizing patients in need of PPC and are willing to start the process. Since most healthcare education systems only recently included PC into the healthcare curriculum, most of the professionals currently working in hospitals are only educated to the extent of self-initiative.

## What is New

- To bridge the existing gap in knowledge, the hospital PC team organized an intervention, presentation, and discussion on PPC, which was offered to all employees in the hospital who are in contact with patients. The personnel’s knowledge, self-assessment of their ability to perform PPC, attitude to participate in PPC, as well as their awareness and understanding of the need for PPC, were evaluated. These four categories have not been tested together before.
- The knowledge, awareness and attitude of the physicians, nurses, and other professionals, improved significantly after the intervention. All the profiles that work together in a team were evaluated simultaneously for the first time.
- However, the self-assessment of their ability to perform PPC did not increase – in fact it decreased. This was unexpected, as existing literature establishes a link between education and quality of PC. Previous experience with the death of a patient has proven to be a stimulus for self-initiative in acquiring knowledge in PPC and was linked with a better attitude and higher awareness of the need for PPC.
- We re-established the importance of education and practical work tailored to the different professional profiles, with adjustments for specific subspecialist areas, especially where patients could be included in early PPC. Although additional studies are needed, we identified the main directions for the further implementation of PPC in clinical practice in our setting.

# Abbreviations

PC palliative care

PPC pediatric palliative care

# Background

According to current guidelines, every child and adolescent living with a life-limiting or life-threatening condition should receive pediatric palliative care (PPC) to alleviate suffering and enhance their quality of life [1–3]. The integration of PPC should become a standard of care for all [2, 4, 5].

There are many barriers and misperceptions in pediatrics which hinder the implementation of PPC, among which the most important is undoubtedly the emotional labor involved in requesting palliative care (PC) [6–8]. Some professionals are concerned about the term ‘palliative care’ itself, feeling its interpretation by families may be negative [9]. Physicians fail to discuss prognosis, because of fear of upsetting the patient and damaging the therapeutic relationship. [10]. Therefore, it is crucial that other professionals, e.g., nurses, who are in closest contact with the family, also know the benefits of PC and are experienced in identifying new patients [11–13].

For the effective implementation of PPC in any environment, it is important to understand what PPC is and what it is not [9, 14]. To improve PC in hospital settings, research and practice have shown that it is first necessary to enable the early identification of patients in need of PC [15]. If healthcare professionals have only a basic knowledge PC, patient involvement may be late, often only in the process of dying [16–18] .

The interdisciplinary hospital PC team at the Children’s Hospital offers PC consultation, help in advance care planning, and advice in providing support to families [19, 20]. Although the hospital PC team is helpful, it is necessary for the medical staff to be able to identify a patient who would benefit from early PC and to request the hospital PC team’s cooperation [21, 22]. Consequently, they all help to ensure that children are exposed to a minimum amount of suffering [5, 23].

In Slovenia, most children with life-limiting or life-threatening conditions are identified at the University Tertiary Children’s Hospital in Ljubljana. A few years ago, a hospital PC team was established, consisting of two pediatric pulmonologists, two pediatric neurologists, a pediatric nephrologist, a clinical geneticist, two registered nurses, and two clinical psychologists. The members of the team are all trained in PPC and have more than 15 years of work experience in their respective fields. In the two years between the team’s inception and the intervention, the team has managed PPC of 70 patients, coordinating different health care providers involved in their care, as well as offering full time support to the families. Soon after the team was established many obstacles in the implementation of PC were recognized. Team members decided to raise awareness of PC’s importance in all departments of the hospital. We considered the recommendations of Eddy et al. [24]: all members of the team participated in the design and implementation of a questionnaire and intervention.

With the study, the hospital PC team aimed to offer all staff members a pediatric-specific orientation in PC appropriate to their roles and responsibilities, and to attract the largest possible number of employees to participate. We also wanted to ascertain the state of the medical staff: knowledge, self-assessment of their ability to perform, attitude towards and awareness of the importance of PC. The objective, based on the findings of the project, was to design starting points for further education and the establishment of accessible, optimal PPC with early involvement of different patients from all departments in the hospital.

# Methods

The medical and paramedical personnel of the institution were invited by the heads of their departments to an intervention, presentation, and discussion on PPC, as well as a survey before and after the intervention. The purpose of the survey was to evaluate four categories: the personnel’s knowledge, self-assessment of their ability to perform PPC, attitude and willingness to participate in PC, as well as awareness and understanding of the need for PPC.

The interventions were conducted by the hospital PPC team and carried out between March 2020 and May 2021 in each hospital department. The lectures were specifically designed for the purpose of the intervention by the team members, who are all experienced lecturers. The lectures were taught by them in Slovenian. The intervention consisted of providing definitions as well as posing provocative questions on what we could do to keep the patients from suffering. It consisted of three parts. The first part covered the physician’s view of the benefits of PC in children and adolescents with life-limiting or life-threatening conditions, and how we can alleviate distress with individually tailored treatment for any of them. In the second part, a nurse demonstrated actions that are easy to perform in the scope of nursing care and provide great relief to both patients and their parents. In the last part, a psychologist presented PPC from the patient's perspective of quality of life and their perception of PPC, presenting various coping mechanisms for serious illness in children and adolescents in different stages of development, and what the child's illness means for the whole family. In all parts, tips were given for better communication with patients, relatives, and within the medical team. An extensive discussion followed at the end of each session. Despite each department receiving a separate presentation, all were of the same content, which was approved by all team members. The lectures were not tested before, to avoid interfering with the results.

The first part of the completely anonymous questionnaire (Supplement 1), designed especially for the project by the hospital PPC team, consisted of demographic questions concerning profession, age, years of experience in pediatrics, experience of a patient dying, and previous participation in PC.

The second part of the questionnaire was aimed at evaluating different areas relating to PC: the healthcare professional’s knowledge (knowledge), self-assessment of their ability to perform PC (self-assessment), attitude and willingness to participate in PC (attitude), as well as their awareness and understanding of the need for PC (awareness). To quantify subjective preferential opinion, thinking and feeling in a scientifically accepted, validated and reliable manner, we used the Likert 5-point symmetrical scale. The questions were formed as statements where participants chose a number between 1 and 5, with number 1 meaning “strongly disagree” and number 5 “strongly agree”. The participants’ mean score of all answers in each of the four categories was calculated and used for further analysis.

The complete anonymity of respondents was ensured in accordance with data protection laws and ethical standards. The results obtained were not linked to any individual or to the department from which data were obtained to prevent the disclosure of participants’ identity.

The results were analyzed using Pandas (version 1.4.2) and SciPy (version 1.8.1) libraries in Python (version 3.10.4; Python Software Foundation, Wilmington, DE, USA).

The Shapiro-Wilks test was used to determine the distribution of data. As most data sets did not follow Gaussian distribution, we used non-parametric tests.

For comparison of results regarding four categories before and after intervention, the Wilcoxon Signed-Rank Test was used. When comparing different groups regarding knowledge, self-assessment, attitude and awareness, the Mann-Whitney U test was used.

# RESULTS

## 1. Demographics

Among all 292 attendees of the intervention, 237 took the survey before and after the intervention. The breakdown of participants by profession is noted in Table 1.

**Table 1** Number and proportions of different professionals among participants

| Broad Category | Profession | Number of participants |
| --- | --- | --- |
| Medical | Physician | 72 (30.4%) |
|  | Nurse | 117 (49.4%) |
|  | Psychologist | 20 (8.4%) |
| Paramedical | Physical therapist | 4 |
|  | Radiology engineer | 3 |
| Other | Dietitian | 4 |
|  | Teacher | 8 |
|  | Kindergarten teacher | 4 |
|  | Other | 5 |
|  |  | 237 (100%) |

**Table 2** Subspecialty breakdown of the participating consultant physicians

| **Department** | **Emergency** | **Neonatology** | **Nephrology** | **Gastroenterology** | **Pulmonology** | **Cardiology** | **Endocrinology** | **Hematology-Oncology** | **Allergology** | **Neurology** | **Pediatric psychiatry** | **ICU** | **Total** |
| --- | --- | --- | --- | --- | --- | --- | --- | --- | --- | --- | --- | --- | --- |
| **Participating consultant physicians** | **1** | **4** | **5** | **4** | **4** | **3** | **6** | **7** | **4** | **2** | **4** | **1** | **45** |

The age structure of participants was as follows: 23.7% (n=55) between 20 and 30 years, 31.9% (n=74) between 31 and 40 years, 20.7% (n=48) between 51 and 60 years, 24.6% (n=57) between 41 and 50 years, and 1.3% (n=3) were older than 60 years.

Among the participants, 35.0% (n=83) had up to 5 years of experience in pediatrics, 16.9% (n=40) had 5 to 15 years of experience, and 46.8% (n=111) had more than 15 years of experience.


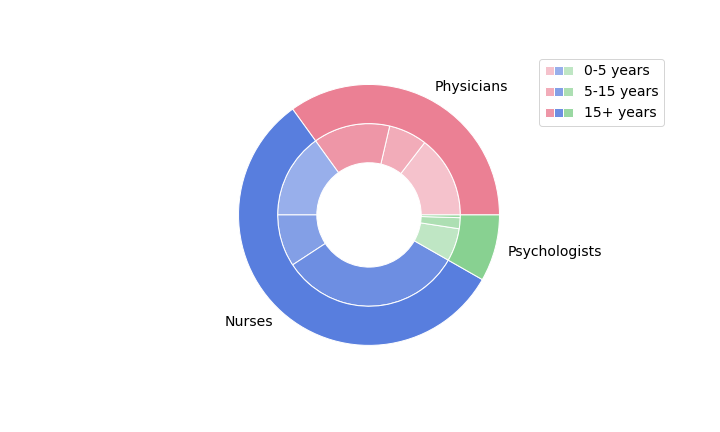


**Fig. 1** Years of experience of the medical professionals

In the group of medical professionals, 41.7% (n = 30) of physicians had less than 5 years of experience in pediatrics, 19.4% (n=14) had 5 to 15 years of experience, and 38.9% (n=28) had more than 15 years of experience. Among nurses, 26.5% (n=31) had less than 5 years of experience in pediatrics, 16.2% (n= 19) had 5 to 15 years of experience, and 57.3% (n=67) had more than 15 years of experience. Among psychologists, 55% (n=11) had less than 5 years of experience, 25% (n=5) had 5 to 15 years of experience, and 5% (n=1) had more than 15 years of experience in pediatrics. 15% (n=3) of psychologists did not answer the question (Figure 1).

There were 69.2% of participants who had already experienced a patient's death: among physicians 70.1%, among nurses 78.6%, and among psychologists 35%. There were 46.4% of participants who reported previous participation in PC: among physicians 40.3%, among nurses 64.1%, and among psychologists 15.0%.

## 2. Comparing all categories before and after the intervention

After the intervention, knowledge was significantly better for all participants combined, but the self-assessment of their ability to perform dropped. Awareness as well as attitude also improved after the intervention (Figure 2).


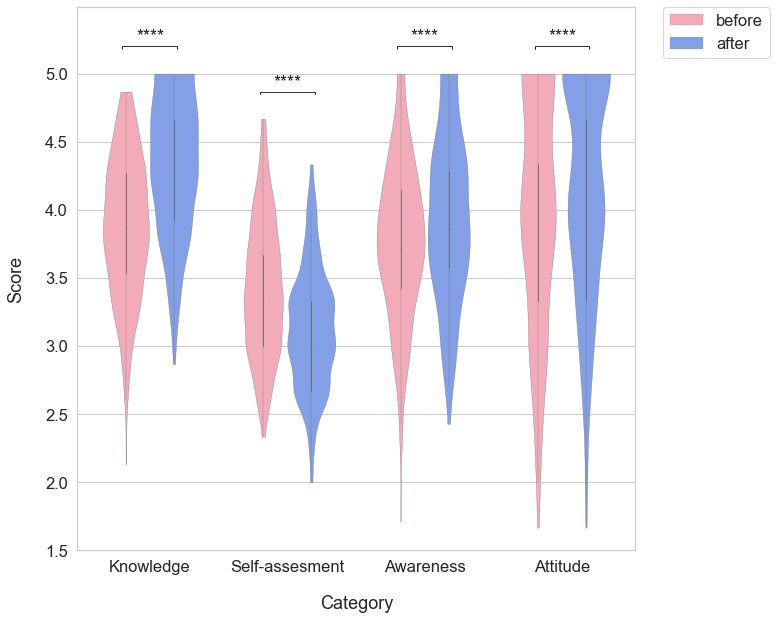


**Fig. 2** Violin plot of scores for different categories before and after the intervention for all participants. NS: non-significant, *p< 0.05, ** p<0.01, *** p<0.001, **** p<0.0001

## 3. Comparison of categories depending on the experience of a patient’s death before and after the intervention

Before the intervention for all participants combined, knowledge, awareness and attitude towards PPC were significantly better in participants who had already experienced the death of a patient, but self-assessment was significantly lower than in participants who had not experienced the death of a patient (Figure 3a). After the intervention, only awareness and attitude were better in participants who had experienced the death of a patient versus participants who had not. Knowledge increased and self-assessment dropped after the intervention but were no longer statistically different between the two groups of participants (Figure 3b).


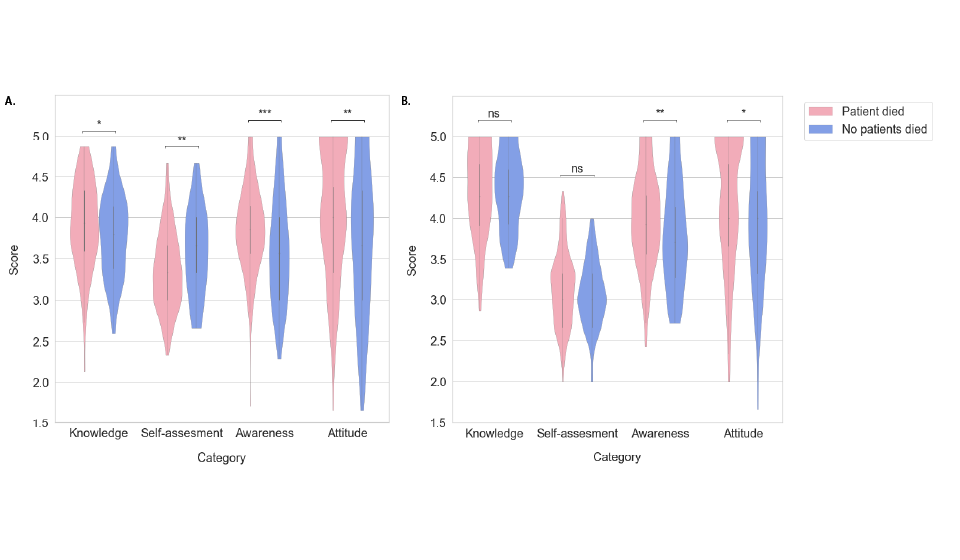


**Fig. 3** Violin plot of scores for different categories before (A) and after (B) the intervention depending on whether the participant experienced the death of a patient. NS: non-significant, *p< 0.05, ** p<0.01, *** p<0.001, **** p<0.0001

We also made comparisons between groups of participants based on age, years of experience, and previous participation in PPC. The only factor that yielded significant differences in all categories was the experience of the death of a patient.

## 4. Comparison of knowledge and self-assessment in the core medical group before and after the intervention

A comparison of knowledge and self-assessment among participants of different professions in the medical group before and after the intervention is shown in Figure 4. Both categories are significantly different in comparing physicians and psychologists versus nurses before and after the intervention.


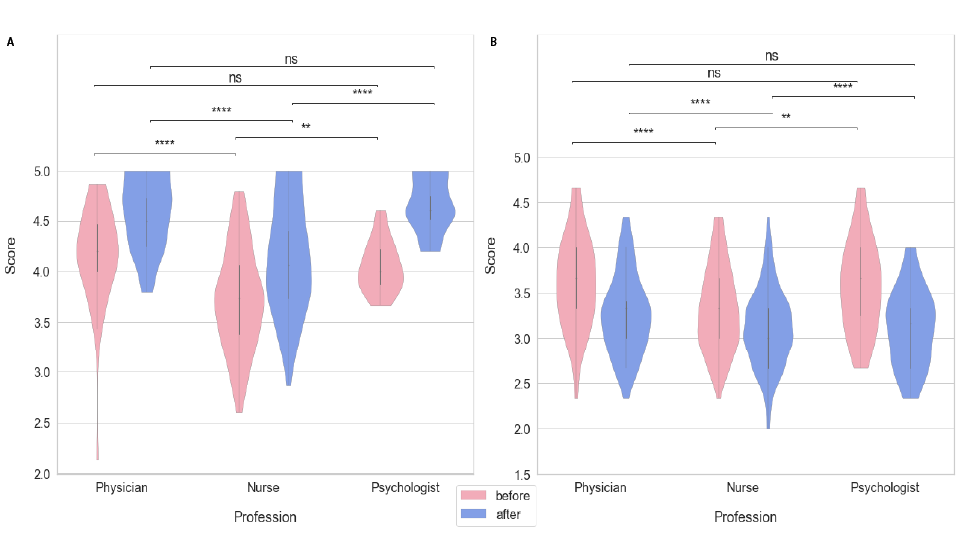


**Fig. 4** Violin plot of knowledge scores (A) and self-assessment scores (B) before and after the intervention for physicians, nurses, and psychologists. NS: non-significant, * p< 0.05, ** p<0.01, *** p<0.001, **** p<0.0001

# Discussion

Given that in our country there is no universal training on PC for healthcare providers, it was expected that the level of knowledge among employees would be relatively low, and that education in improving attitude and awareness would be needed for all personnel. At the same time, we realized that for further development, it would be necessary to determine the existing conditions in our hospital. We were not able to find a study in which these categories, including self-assessment of ability to perform PC, attitude, and awareness, were studied in medical professionals of different profiles who work together in a tertiary children's hospital in different subspecialties. Tsao et al. measured baseline palliative knowledge, attitudes, and self-assessment, however only with physicians from oncology and general adult care [25]. We found some similarities with the study of Newton and Sebbens, which took place in several departments in a tertiary care pediatric facility where the effect of educational sessions on hospital-wide referral rates and provider comfortability after survey was studied. Although the study differed with respect to participant professions, the results are comparable with ours by the positive effect of educational sessions demonstrated. With knowledge and experience, awareness of the need for PC is improved and aversion to work in PC is lessened [26]. Ghoshal et al. evaluated attitude and knowledge along with skills and practice after modules on PPC had been uniformly conducted among healthcare workers in PPC facilities [27]. Physicians and nurses had a better level of knowledge and attitude than social workers and counselors.

It was also clear from the beginning that only a single intervention would not be sufficient for healthcare providers to achieve the satisfactory implementation of PC in hospitals. However, this might prompt the staff to start thinking about PC, become interested in identifying patients who would benefit from PC, and enhance their awareness and attitude of the importance of PC, which may have an important influence on the quality of care they provide [28, 29].

After the intervention, employees answered questions about the PC content more correctly, were more willing to participate in PC, and were more aware of the importance of PC. The result, namely, that the knowledge, attitude, and awareness of all participants increased, is favorable and expected. It is, however, unexpected that the self-assessment of their ability to perform PC did not increase with the intervention.

It is possible that after learning about PC the participants realized that they were not equipped to provide this care and therefore felt less able to provide it themselves. This, combined with the increase in their awareness and attitude scores, might lead them to seek further education more actively, attending additional lectures and practical workshops, which is a desired outcome.

The only studied factor that yielded significant differences in all categories was the experience of the death of a patient. Apparently, the experience of a patient dying is an important driver for acquiring knowledge, attitude and understanding of the importance of PC, but not for feeling the ability to perform it, which is interesting. It was surprising to learn that those participants who had experienced the death of a patient before the intervention expressed feeling less able to perform PC compared to the participants who had not had this experience. Perhaps the latter were not aware of the complexity of PC and the emotional burden.

The results of a comparison of self-assessment in the two groups of participants after the intervention, which showed that the difference in the self-assessment of their ability to perform PPC after the intervention had disappeared, despite having decreased in both groups, may suggest an explanation for such an outcome. The intervention revealed the difficulty of working with seriously ill children and possibly discouraged the participants, which is the opposite of what we wanted to achieve. Another possibility is that lecturers overemphasized the work of the hospital PC team, causing participants to misunderstand that PC was mainly the obligation of the hospital PC team and not of all employees. Though it is undoubtedly important for the hospital PC team to offer consultation, it should bring home the message that the departmental medical teams need to provide PPC to patients themselves in their daily work.

We can self-critically assess that this intervention alone may not improve the implementation of PC in our environment to the desired extent. It is, however, optimistic that the intervention has helped to increase the participants’ knowledge, attitude, and awareness of the importance of PC. The results showing a decrease in self-assessment of ability to perform PC after the intervention present a clear direction for further research and intervention: what are the reasons for the feeling of inability to perform, also by additionally defining the feelings of those who have already had the experience of a patient dying, and later designing interventions to help overcome these reservations.

The interventions were designed to come closer to the different healthcare professionals’ profiles within each department. They were carried out for all professionals in one department at a time to show them that the work of different profiles is equally important when they work as a medical team in PPC.

Knowledge is difficult to evaluate [30]. Comparing knowledge between different profiles of professionals is especially demanding. Most of the questions in the knowledge category were general statements about what PC is and what it is not. Both physicians and psychologists scored better than nurses in knowledge, which surprised us, especially because the significant difference remained even after education. We infer that our intervention was not sufficiently tailored to the needs of different profiles. The level of knowledge was an insight into the extent of the incorrect notion that PC is simply caring for a dying patient. Even though knowledge increased after the intervention, self-assessment worsened. Since nurses tend to have more constant contact with patients and their families, and are sometimes easier to approach with questions, it is crucial they have the knowledge and feel empowered to give advice and talk about early involvement in PPC. We realized that it might be difficult for nurses to identify patients who would benefit from early PPC, since their knowledge scores were lower and they had a significantly greater feeling of inadequate knowledge, when compared to other professionals. [12–15, 21, 31–34].

There are several limitations to our study because there is no standardized education material about PC for healthcare providers in our country. No similar research with different professional profiles included in the same tertiary-level pediatric hospital has been found with which we could compare our results. Finally, the survey was designed specifically for the intervention and has not been validated in other environments.

# conclusions

The knowledge, awareness and attitude of physicians, nurses, and other professionals in the tertiary- level pediatric hospital improved significantly after the presentation and discussion on PPC provided by the hospital PC team. Previous experience of a patient dying has proven to be a stimulus for self-initiative in acquiring knowledge in PC and improving attitude and awareness towards PC.

We believe that the intervention presents an important first step of PPC implementation. Non-palliative care professionals might be more willing to consult the hospital PC team and talk about PC with their patients, even if they do not feel confident enough to provide it themselves, resulting in more frequent and timely referrals.

In further efforts to improve the implementation of PC in our environment, there are many challenges. More education and practical work tailored to the different professional profiles are needed, different educational components for training practical skills (e.g. roleplaying) and other adjustments should be used, especially for the subspecialist areas where patients could be included in early PC.

# REFERENCES

1. Vision, Mission & Aims. In: ICPCN. https://www.icpcn.org/about-icpcn/vision-mission-aims/. Accessed 1 Aug 2022
2. Mack JW, Wolfe J (2006) Early integration of pediatric palliative care: for some children, palliative care starts at diagnosis. Curr Opin Pediatr 18:10–14. https://doi.org/10.1097/01.mop.0000193266.86129.47
3. Bergstraesser E (2013) Pediatric palliative care-when quality of life becomes the main focus of treatment. Eur J Pediatr 172:139–150. https://doi.org/10.1007/s00431-012-1710-z
4. (2000) American Academy of Pediatrics. Committee on Bioethics and Committee on Hospital Care. Palliative care for children. Pediatrics 106:351–357
5. SECTION ON HOSPICE AND PALLIATIVE MEDICINE AND COMMITTEE ON HOSPITAL CARE (2013) Pediatric Palliative Care and Hospice Care Commitments, Guidelines, and Recommendations. Pediatrics 132:966–972. https://doi.org/10.1542/peds.2013-2731
6. Neuburg L (2021) Early Initiation of Pediatric Palliative Care. J Pediatr Health Care Off Publ Natl Assoc Pediatr Nurse Assoc Pract 35:114–119. https://doi.org/10.1016/j.pedhc.2020.05.006
7. Friedrichsdorf SJ (2017) Contemporary Pediatric Palliative Care: Myths and Barriers to Integration into Clinical Care. Curr Pediatr Rev 13:8–12. https://doi.org/10.2174/1573396313666161116101518
8. Szymczak JE, Schall T, Hill DL, Walter JK, Parikh S, DiDomenico C, Feudtner C (2018) Pediatric Oncology Providers’ Perceptions of a Palliative Care Service: The Influence of Emotional Esteem and Emotional Labor. J Pain Symptom Manage 55:1260–1268. https://doi.org/10.1016/j.jpainsymman.2018.01.019
9. Morstad Boldt A, Yusuf F, Himelstein BP (2006) Perceptions of the term palliative care. J Palliat Med 9:1128–1136. https://doi.org/10.1089/jpm.2006.9.1128
10. Davies B, Sehring SA, Partridge JC, Cooper BA, Hughes A, Philp JC, Amidi-Nouri A, Kramer RF (2008) Barriers to palliative care for children: perceptions of pediatric health care providers. Pediatrics 121:282–288. https://doi.org/10.1542/peds.2006-3153
11. Pearson H (2010) Managing the emotional aspects of end of life care for children and young people. Paediatr Nurs 22:31–35. https://doi.org/10.7748/paed2010.09.22.7.31.c7951
12. Feeg VD, Elebiary H (2005) Exploratory study on end-of-life issues: barriers to palliative care and advance directives. Am J Hosp Palliat Care 22:119–124. https://doi.org/10.1177/104990910502200207
13. World Health Organization Palliative care definition. https://www.who.int/health-topics/palliative-care. Accessed 1 Aug 2022
14. Thacker KS (2008) Nurses’ advocacy behaviors in end-of-life nursing care. Nurs Ethics 15:174–185. https://doi.org/10.1177/0969733007086015
15. Borowske D (2012) Straddling the fence: ICU nurses advocating for hospice care. Crit Care Nurs Clin North Am 24:105–116. https://doi.org/10.1016/j.ccell.2012.01.006
16. Le BHC, Mileshkin L, Doan K, Saward D, Spruyt O, Yoong J, Gunawardana D, Conron M, Philip J (2014) Acceptability of early integration of palliative care in patients with incurable lung cancer. J Palliat Med 17:553–558. https://doi.org/10.1089/jpm.2013.0473
17. Thompson LA, Knapp C, Madden V, Shenkman E (2009) Pediatricians’ perceptions of and preferred timing for pediatric palliative care. Pediatrics 123:e777-782. https://doi.org/10.1542/peds.2008-2721
18. Nyirő J, Zörgő S, Enikő F, Hegedűs K, Hauser P (2018) The timing and circumstances of the implementation of pediatric palliative care in Hungarian pediatric oncology. Eur J Pediatr 177:1173–1179. https://doi.org/10.1007/s00431-018-3170-6
19. Delgado-Corcoran C, Bennett EE, Bodily SA, Wawrzynski SE, Green D, Moore D, Cook LJ, Olson LM (2021) Prevalence of specialised palliative care consultation for eligible children within a paediatric cardiac ICU. Cardiol Young 31:1458–1464. https://doi.org/10.1017/S1047951121000433
20. Bergstraesser E, Inglin S, Abbruzzese R, Marfurt-Russenberger K, Hošek M, Hornung R (2013) The needs of professionals in the palliative care of children and adolescents. Eur J Pediatr 172:111–118. https://doi.org/10.1007/s00431-012-1880-8
21. Pelant D, McCaffrey T, Beckel J (2012) Development and implementation of a pediatric palliative care program. J Pediatr Nurs 27:394–401. https://doi.org/10.1016/j.pedn.2011.06.005
22. World Health Organization (2016) Planning and implementing palliative care services: a guide for programme managers, 2e éd. Organisation mondiale de la Santé, Genève
23. Drake R (2018) Palliative Care for Children in Hospital: Essential Roles. Children 5:26. https://doi.org/10.3390/children5020026
24. Eddy K, Jordan Z, Stephenson M (2016) Health professionals’ experience of teamwork education in acute hospital settings: a systematic review of qualitative literature. JBI Database Syst Rev Implement Rep 14:96–137. https://doi.org/10.11124/JBISRIR-2016-1843
25. Tsao L, Slater SE, Doyle KP, Cuong DD, Khanh QT, Maurer R, Thy DNM, Thinh DHQ, Tuan TD, Dung DV, Khue LN, Krakauer EL (2019) Palliative Care–Related Knowledge, Attitudes, and Self-Assessment Among Physicians in Vietnam. J Pain Symptom Manage 58:1015-1022.e10. https://doi.org/10.1016/j.jpainsymman.2019.08.001
26. Newton K, Sebbens D (2020) The Impact of Provider Education on Pediatric Palliative Care Referral. J Pediatr Health Care Off Publ Natl Assoc Pediatr Nurse Assoc Pract 34:99–108. https://doi.org/10.1016/j.pedhc.2019.07.007
27. Ghoshal A, Talawadekar P, Palleri A, Marston J, Muckaden M (2018) Impact of Educational Training in Improving Skills, Practice, Attitude, and Knowledge of Healthcare Workers in Pediatric Palliative Care: Children’s Palliative Care Project in the Indian State of Maharashtra. Indian J Palliat Care 24:411–425. https://doi.org/10.4103/IJPC.IJPC_43_18
28. Braun M, Gordon D, Uziely B (2010) Associations between oncology nurses’ attitudes toward death and caring for dying patients. Oncol Nurs Forum 37:E43-49. https://doi.org/10.1188/10.ONF.E43-E49
29. Rusalen F, Ferrante A, Pò C, Salata M, Agosto C, Benini F (2014) Pain therapy, pediatric palliative care and end-of-life care: training, experience, and reactions of pediatric residents in Italy. Eur J Pediatr 173:. https://doi.org/10.1007/s00431-014-2304-8
30. Li WW, Chhabra J, Singh S (2021) Palliative care education and its effectiveness: a systematic review. Public Health 194:96–108. https://doi.org/10.1016/j.puhe.2021.02.033
31. Erikson A, Davies B (2017) Maintaining Integrity: How Nurses Navigate Boundaries in Pediatric Palliative Care. J Pediatr Nurs 35:42–49. https://doi.org/10.1016/j.pedn.2017.02.031
32. Lindley LC, Cozad MJ (2017) Nurse Knowledge, Work Environment, and Turnover in Highly Specialized Pediatric End-of-Life Care. Am J Hosp Palliat Care 34:577–583. https://doi.org/10.1177/1049909116649415
33. O’Shea ER, Mager D (2019) End-of-life nursing education: Enhancing nurse knowledge and attitudes. Appl Nurs Res ANR 50:151197. https://doi.org/10.1016/j.apnr.2019.151197
34. Abuhammad S, Almasri R (2022) Impact of educational programs on nurses’ knowledge and attitude toward pediatric palliative care. Palliat Support Care 20:397–406. https://doi.org/10.1017/S1478951521001139

# Supplement 1

^*^The questions were classified as follows: evaluation of healthcare professional’s knowledge: question nos. 2, 3, 5-8, 10, 16, 19-22, 25-27 (strongly agree was translated to 5 points when the statement was true, and the scoring was reversed for statements that were untrue); self-assessment of ability to perform PC: question nos. 1, 18, 28 (value 1 for the poorest self-assessment and value 5 for the best assessment; strongly agree was translated to 5 points when the statement expressed sufficient knowledge to be able to perform PC, and the scoring was reversed for statements expressing not enough capacity to perform PC); attitude and willingness to participate in PC: question nos. 4, 13, 24 (the most expressed attitude was 5); awareness and understanding of the need for PC: question nos. 9, 11, 12, 14, 15, 17, 23 (the most expressed awareness was 5).

**Questionnaire**

Your personal password:

*It is important to remember this password and use the same one on both questionnaires*

|  |  |  |  |  |  |  |  |  |  |
| --- | --- | --- | --- | --- | --- | --- | --- | --- | --- |
| *1* | *2* | *3* | *4* | *5* | *6* | *7* | *8* | *9* | *10* |

1. Gender: M F

2. How old are you?

A. 20-30 years

B. 31-40 years

C. 41-50 years

D. 51-60 years

E. more than 60 years

3. What is your profession?

A. Nurse

B. Medical doctor

C. Psychologist

D. Other: _______________________

4. How long have you been employed at the Pediatric Clinic?

A. Less than a year

B. 1 to 5 years

C. 5 to 10 years

D. 10 to 15 years

E. 15 to 25 years

F. More than 25 years

5. How long have you been practicing your profession in pediatrics?

A. Less than a year

B. 1 to 5 years

C. 5 to 10 years

D. 10 to 15 years

E. 15 to 25 years

F. More than 25 years

6. What is your subspeciality area?

A. Admission and triage ward

B. Neonatology

C. Nephrology

D. Gastroenterology, Hepatology and Nutrition

E. Pulmonary Disease

F. Cardiology

G. Endocrinology, Diabetes and Metabolic diseases

H. Pediatric Hematology and Oncology

I. Allergology, Rheumatology and Clinical Immunology

J. Pediatric, Adolescent and Developmental Neurology

K. Pediatric psychiatry

L. Radiology

7. Have you ever provided palliative care?

A. Yes

B. No

8. Have you experienced the death of a patient?

A. Yes

B. No

9. Choose the answer that best describes your opinion about each statement:

|  |  | Strongly disagree | Disagree | Neither agree nor disagree | Agree | Strongly agree |
| --- | --- | --- | --- | --- | --- | --- |
| 1. | I don't know enough about palliative care. | 1 | 2 | 3 | 4 | 5 |
| 2. | The decision about including a patient in palliative care is made in the weeks before the patient’s death. | 1 | 2 | 3 | 4 | 5 |
| 3. | Patients included in palliative care will not live much longer. | 1 | 2 | 3 | 4 | 5 |
| 4. | I am afraid of being involved in providing palliative care. | 1 | 2 | 3 | 4 | 5 |
| 5. | Palliative care is not treatment but merely easing the suffering of the patient in the days before their death. | 1 | 2 | 3 | 4 | 5 |
| 6. | Palliative care includes support for the family spanning beyond the death of the child. | 1 | 2 | 3 | 4 | 5 |
| 7. | Morphine preparations must not be given to the patient when difficulties with breathing occur before death. | 1 | 2 | 3 | 4 | 5 |
| 8. | Once the patient is included in palliative care, we stop all treatment except analgesia. | 1 | 2 | 3 | 4 | 5 |
| 9. | Determining whether a child needs palliative care is the role of the palliative team. | 1 | 2 | 3 | 4 | 5 |
| 10. | The core principle of palliative care is the decision to limit treatment. | 1 | 2 | 3 | 4 | 5 |
| 11. | We don't treat children that would require palliative care at our ward. | 1 | 2 | 3 | 4 | 5 |
| 12. | Education about palliative care is important for what I do. | 1 | 2 | 3 | 4 | 5 |
| 13. | I feel uncomfortable talking about palliative care of children and adolescents. | 1 | 2 | 3 | 4 | 5 |
| 14. | Specific knowledge and skills are needed to manage a palliative care patient. | 1 | 2 | 3 | 4 | 5 |
| 15. | Palliative care patients should not be treated at our ward because they have a negative influence on the treatment of other patients. | 1 | 2 | 3 | 4 | 5 |
| 16. | We consider the wishes of the patient and their family when planning palliative care. | 1 | 2 | 3 | 4 | 5 |
| 17. | I work with children who would benefit from inclusion in palliative care. | 1 | 2 | 3 | 4 | 5 |
| 18. | I have enough knowledge about palliative care to carry out my work. | 1 | 2 | 3 | 4 | 5 |
| 19. | Inclusion in palliative care means that we have given up on the patient. | 1 | 2 | 3 | 4 | 5 |
| 20. | The most important thing is that the patient is not aware of anything before death. | 1 | 2 | 3 | 4 | 5 |
| 21. | Palliative care means alleviating the experience of death. | 1 | 2 | 3 | 4 | 5 |
| 22. | Good communication skills are required to provide palliative care. | 1 | 2 | 3 | 4 | 5 |
| 23. | Palliative care requires separate personnel and accommodation. | 1 | 2 | 3 | 4 | 5 |
| 24. | I don't want to be part of palliative care because it is mostly linked to suffering. | 1 | 2 | 3 | 4 | 5 |
| 25. | Palliative care is a branch of medicine that provides a higher quality of life. | 1 | 2 | 3 | 4 | 5 |
| 26. | Using morphine-based preparations accelerates the onset of death. | 1 | 2 | 3 | 4 | 5 |
| 27. | Patients in palliative care are no longer fed. | 1 | 2 | 3 | 4 | 5 |
| 28. | I do not know how to recognize a patient that needs palliative care. | 1 | 2 | 3 | 4 | 5 |
