# Supplementary material for: Challenges in establishing optimal pediatric palliative care at the university hospital in Slovenia
Source: Eur J Pediatr. 2023 Jan 21;182(3):1393–401. doi: 10.1007/s00431-023-04806-7 (PMC10023644; doi:10.1007/s00431-023-04806-7)
Supplement: Supplementary file 2 — Supplementary file2 (PDF 104 KB) [file 431_2023_4806_MOESM2_ESM.pdf]

SUPPLEMENT 1

*The questions were classified as follows: evaluation of healthcare professional’s knowledge: question nos. 2, 3, 5-8, 10, 16, 19-22, 25-27 (strongly agree was translated to 5 points when the statement was true, and the scoring was reversed for statements that were untrue); self-assessment of ability to perform PC: question nos. 1, 18, 28 (value 1 for the poorest self-assessment and value 5 for the best assessment; strongly agree was translated to 5 points when the statement expressed sufficient knowledge to be able to perform PC, and the scoring was reversed for statements expressing not enough capacity to perform PC); attitude and willingness to participate in PC: question nos. 4, 13, 24 (the most expressed attitude was 5); awareness and understanding of the need for PC: question nos. 9, 11, 12, 14, 15, 17, 23 (the most expressed awareness was 5).

Questionnaire

Your personal password:

It is important to remember this password and use the same one on both questionnaires

1	*2*	*3*	*4*	*5*	*6*	*7*	*8*	*9*	*10*

1. Gender: M F

2. How old are you?

A. 20-30 years
B. 31-40 years
C. 41-50 years
D. 51-60 years
E. more than 60 years

3. What is your profession?

A. Nurse
B. Medical doctor
C. Psychologist
D. Other: ________________________

4. How long have you been employed at the Pediatric Clinic?

A. Less than a year
B. 1 to 5 years
C. 5 to 10 years
D. 10 to 15 years
E. 15 to 25 years
F. More than 25 years

5. How long have you been practicing your profession in pediatrics?

A. Less than a year
B. 1 to 5 years
C. 5 to 10 years
D. 10 to 15 years
E. 15 to 25 years
F. More than 25 years

6. What is your subspeciality area?

A. Admission and triage ward
B. Neonatology
C. Nephrology
D. Gastroenterology, Hepatology and Nutrition
E. Pulmonary Disease
F. Cardiology
G. Endocrinology, Diabetes and Metabolic diseases
H. Pediatric Hematology and Oncology
I. Allergology, Rheumatology and Clinical Immunology
J. Pediatric, Adolescent and Developmental Neurology
K. Pediatric psychiatry
L. Radiology

7. Have you ever provided palliative care?

A. Yes
B. No

8. Have you experienced the death of a patient?

A. Yes
B. No

9. Choose the answer that best describes your opinion about each statement:

		Strongly disagree	Disagree	Neither agree nor disagree	Agree	Strongly agree
1.	I don't know enough about palliative care.	1	2	3	4	5
2.	The decision about including a patient in palliative care is made in the weeks before the patient’s death.	1	2	3	4	5
3.	Patients included in palliative care will not live much longer.	1	2	3	4	5

4.	I am afraid of being involved in providing palliative care.	1	2	3	4	5
5.	Palliative care is not treatment but merely easing the suffering of the patient in the days before their death.	1	2	3	4	5
6.	Palliative care includes support for the family spanning beyond the death of the child.	1	2	3	4	5
7.	Morphine preparations must not be given to the patient when difficulties with breathing occur before death.	1	2	3	4	5
8.	Once the patient is included in palliative care, we stop all treatment except analgesia.	1	2	3	4	5
9.	Determining whether a child needs palliative care is the role of the palliative team.	1	2	3	4	5
10.	The core principle of palliative care is the decision to limit treatment.	1	2	3	4	5
11.	We don't treat children that would require palliative care at our ward.	1	2	3	4	5
12.	Education about palliative care is important for what I do.	1	2	3	4	5
13.	I feel uncomfortable talking about palliative care of children and adolescents.	1	2	3	4	5
14.	Specific knowledge and skills are needed to manage a palliative care patient.	1	2	3	4	5
15.	Palliative care patients should not be treated at our ward because they have a negative influence on the treatment of other patients.	1	2	3	4	5
16.	We consider the wishes of the patient and their family when planning palliative care.	1	2	3	4	5
17.	I work with children who would benefit from inclusion in palliative care.	1	2	3	4	5
18.	I have enough knowledge about palliative care to carry out my work.	1	2	3	4	5

19.	Inclusion in palliative care means that we have given up on the patient.	1	2	3	4	5
20.	The most important thing is that the patient is not aware of anything before death.	1	2	3	4	5
21.	Palliative care means alleviating the experience of death.	1	2	3	4	5
22.	Good communication skills are required to provide palliative care.	1	2	3	4	5
23.	Palliative care requires separate personnel and accommodation.	1	2	3	4	5
24.	I don't want to be part of palliative care because it is mostly linked to suffering.	1	2	3	4	5
25.	Palliative care is a branch of medicine that provides a higher quality of life.	1	2	3	4	5
26.	Using morphine-based preparations accelerates the onset of death.	1	2	3	4	5
27.	Patients in palliative care are no longer fed.	1	2	3	4	5
28.	I do not know how to recognize a patient that needs palliative care.	1	2	3	4	5
